# Strategic Advances in Targeted Delivery Carriers for Therapeutic Cancer Vaccines

**DOI:** 10.3390/ijms26146879

**Published:** 2025-07-17

**Authors:** Junxi Wu, Jinghui Liang, Yuan Zhang, Chunyan Dong, Dejiang Tan, Hongyu Wang, Yiyang Zheng, Qing He

**Affiliations:** State Key Laboratory of Drug Regulatory Sciences, National Institutes for Food and Drug Control, Beijing 102629, China; junxiwu722@outlook.com (J.W.); liangjinghui100@163.com (J.L.); zhyuan_cn@126.com (Y.Z.); dongchunyan@nifdc.org.cn (C.D.); tandj@nifdc.org.cn (D.T.); wanghongyu@stu.cpu.edu.cn (H.W.); 15195222136@163.com (Y.Z.)

**Keywords:** therapeutic cancer vaccines, targeted delivery systems, active and passive targeting, viral and non-viral vectors

## Abstract

Cancer is one of the major global health burdens, and more effective treatments are needed. At present, there are surgery, targeted therapy, and immunotherapy for the treatment of tumors, but due to the limitations of diagnostic technology and drug resistance, surgery and targeted therapy have little effect. Active immunization in the field of immunotherapy can mobilize host immunity, trigger tumor-specific T-cell responses, and produce targeted cytotoxicity. Its efficacy largely depends on the targeted delivery efficiency of cancer vaccines. Although immunotherapy is more durable than other approaches, immunosuppression in the tumor microenvironment and immune evasion by malignant cells limit the therapeutic efficacy of cancer vaccines. To overcome these challenges, this review summarizes key strategies for improving vaccine vector targeting, as well as recent advances and trends in delivery systems.

## 1. Introduction

Cancer is a leading cause of global mortality, with over 20 million new cases and 9.7 million deaths reported in 2022. In the U.S., it ranks as the second leading cause of death, with projections estimating 2.04 million new cases and 610,000 deaths by 2025 [1,2]. Current treatments include surgery, radiotherapy, chemotherapy, and immunotherapy. Surgery is mainly aimed at early-stage tumors, but it is limited by diagnostic methods. Chemotherapy and radiotherapy are common treatments, but further progression is limited by systemic toxic effects and limited efficacy in advanced or recurrent disease [3,4,5]. The evolution of cancer treatments—from radical surgery and cytotoxic chemotherapy to immune-based therapies—has been well documented by the American Cancer Society (ACS) [6], providing the historical context for the emergence of therapeutic cancer vaccines. The limitations of these approaches have shifted attention to immunotherapy strategies, particularly cancer vaccines that are designed to stimulate tumor-specific immune responses.

The concept of cancer vaccination can be traced back to the late 19th century, when Dr. William B. Coley observed tumor regression in sarcoma patients following streptococcal infections. He hypothesized that the infection-induced immune activation was responsible for the antitumor effects [7]. In the mid-20th century, the introduction of the theory of immune surveillance provided a mechanistic basis for immune-based tumor control. This period saw a surge in research and clinical trials of immune strategies, laying the groundwork for the development of therapeutic cancer vaccines, and in 2010, the FDA approved the first therapeutic cancer vaccine, Sipuleucel-T, a milestone that catalyzed a new era of personalized cancer immunotherapy [8,9]. The cancer vaccines can be classified into preventive vaccines and therapeutic vaccines. The former prevents the occurrence of virus-related tumors by inducing antiviral immunity; the latter aims to activate tumor antigen-specific T cells to clear the formed tumors [10]. Preventive vaccines mainly achieve tumor prevention through antiviral effects, such as human papillomavirus (HPV)- and hepatitis B virus (HBV)-related cancers. In contrast, therapeutic vaccines rely on the activation of antigen-presenting cells (APCs), especially dendritic cells, to promote effector and memory T-cell responses. For example, Sipuleucel-T, which has been approved for the treatment of prostate cancer, is a typical example of therapeutic vaccines [9,11,12]. Despite their specificity, safety, and long-term memory induction, the clinical efficacy of cancer vaccines remains limited by several factors. These factors include an immunosuppressive tumor microenvironment (TME), delayed onset of immune activation, and strong dependence on adjuvants and delivery platforms. Cancer vaccines can be broadly categorized into four platforms based on the mode of antigen presentation: cellular vaccines, protein/peptide vaccines, whole-tumor-cell vaccines, and nucleic acid vaccines. Cellular vaccines (including dendritic cell vaccines) are highly personalized but are complex, costly, and time-consuming to produce. Protein/peptide vaccines are usually safe and easy to synthesize but are weakly immunogenic and susceptible to enzymatic degradation in vivo. Whole-tumor-cell vaccines have a broad antigenic spectrum but present challenges in quality control and batch standardization. Nucleic acid vaccines, especially mRNA-based formulations, offer a high degree of flexibility and the potential for rapid design, but their efficacy is closely linked to efficient intracellular delivery and strong innate immune activation. Among these platforms, nucleic acid vaccines—particularly mRNA-based formulations—stand out for their rapid design flexibility and multi-antigen encoding capability. However, their therapeutic efficacy remains critically dependent on efficient delivery systems and immunostimulatory adjuvants. Therefore, the development of robust, targeted delivery platforms has become central to improving nucleic acid vaccine performance. The following sections of this review will discuss recent advances in such delivery systems, which can be broadly categorized into viral and non-viral types [13,14,15,16,17].

In addition to the influence of vector, vaccine efficacy is affected by many factors. For example, antigen selection determines specificity and immunogenicity, and vector and adjuvant designs affect the degree of antigen presentation and response [18]. These aspects, including antigen design, immunogenicity optimization, production cost, and quality control, have been extensively reviewed elsewhere [17,19,20] and are not the focus of this review. Here, we focus specifically on delivery strategies to enhance the therapeutic efficacy of cancer vaccines. At the same time, immunosuppression in the TME, immune evasion mechanisms such as antigenic heterogeneity and high mutational burden, and inefficient delivery systems remain major obstacles to clinical translation [10,19,21,22,23,24]. Targeted vectors are an important strategy to overcome these obstacles. Properly designed vectors can also reprogram tumor tissues and organs and synergize with approaches against tumor immune escape. Systematic evaluation of vector platforms and the mechanistic basis of their targeting is essential to advance the development of next-generation vaccines.

**Figure 1:**
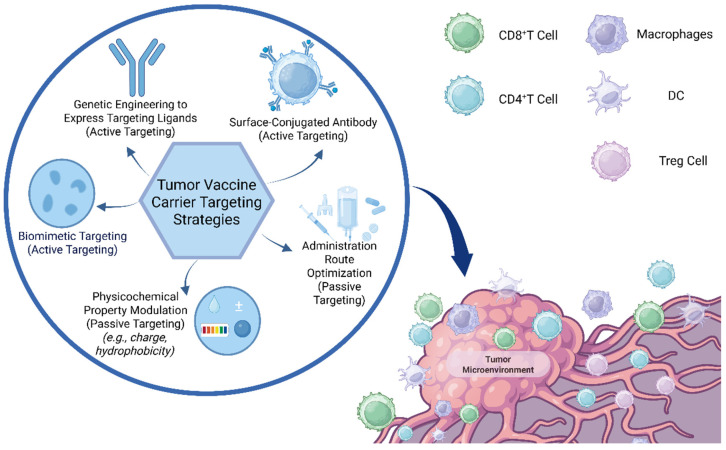
Schematic of vector targeting strategies, illustrating genetic engineering, surface modification, and biomembrane-based targeting approaches. Created in https://BioRender.com.

This review focuses on recent advances in targeting strategies for viral and non-viral vaccine delivery platforms. These include active targeting approaches—such as antibody conjugation, genetic ligand expression, and biomimetic engineering—as well as passive strategies involving physicochemical property modulation. As illustrated in Figure 1, these strategies aim to enhance vaccine accumulation within the TME and promote engagement with antigen-presenting cells and T lymphocyte subsets. This conceptual framework provides a mechanistic basis for subsequent discussions of vector design and tumor specificity.

## 2. Viral Vector Vaccines

Viral vectors are an important part of cancer vaccine delivery due to their efficient gene transfer and immunogenicity. Viral platforms such as adenovirus (Ad), poxvirus, and adeno-associated virus (AAV) have been genetically engineered to encode tumor-associated or tumor-specific antigens (TAA/TSA), enabling precise in situ antigen expression [25]. In addition to antigen payload, recent studies have focused on modifying viral capsid or envelope proteins to improve the cell tropism and tissue-specific uptake of vectors, thereby enhancing the ability of vectors to target lymphoid organs or TME. These advances not only enhance the antigen presentation ability of dendritic cells but also facilitate large-scale production and clinical translation [26].

### 2.1. Adenoviral (Ad) Vectors

Ad are non-enveloped, double-stranded DNA viruses with icosahedral symmetry and high transduction efficiency. Their genomes remain episomal, minimizing the risk of insertional mutagenesis. Since the 1990s, Ad vectors have been widely employed in gene therapy, vaccine development, and genome editing [27,28,29,30]. However, several limitations hinder clinical application.

The prevalence of pre-neutralizing antibodies against common Ad serotypes in the population reduces vaccine efficacy. To address this problem, alternative serotypes with low seroprevalence have been developed, such as those from non-primate hosts, or variants that have undergone capsid design to evade immune memory [31,32,33]. In addition, Ads exhibit a strong hepatotropism, leading to liver accumulation and toxicity, thereby affecting treatment efficiency. In 2020, Lu Z.H. and colleagues reported that wild-type gorilla adenovirus (GAd) exhibits intrinsic lung tropism. By adding a myeloid cell-binding peptide (MBP) to the coat of the virus, they changed the tissue distribution of the virus, reduced the lung targeting, and allowed the vector to be used in more scenarios [34]. Combining natural vector tropism with ligand-based modification of capsid proteins is a strategy to circumvent immunological barriers and improve tissue-specific delivery.

Among adenovirus platforms, recombinant Ad vectors, both replication-deficient and conditionally replicating, have attracted the attention of researchers. The natural tumor tropism of conditionally replicating adenoviruses (oncolytic adenoviruses, OAds) has been a key tool in the design of cancer vaccines [35]. For example, capsid fusion with a soluble TRAIL ligand containing a leucine zipper has been shown to enhance OAd targeting of TRAIL-expressing tumors, improving therapeutic specificity and potency [36]. Although most cancer vaccines focus on encoding TAA or TSA to elicit T-cell responses, vector targeting remains a critical factor for immunogenicity and safety. Local enrichment of Ad vectors within the tumor could enhance site-specific immunity while minimizing systemic toxicity.

Strategies to enhance Ad targeting can be divided into two broad categories: capsid protein modification and genetic engineering. Approaches based on capsid-protein modification include conjugated targeting ligands, incorporation of tumor chemokines, or structural changes in fibrin to increase tissue-specific affinity [37,38,39]. Conversely, genetic engineering may delete essential replicating genes to limit viral spread to tumor cells or insert genes encoding tumor-targeting molecules [40,41,42,43]. Physical modifications of coat proteins, such as covalent binding, provide rapid and transient anti-tumor immunity to vectors, offering a flexible strategy for short-term immunotherapy and exploratory applications. In contrast, genetically engineered surface modifications allow for sustained, controlled expression of the ligand and are therefore more suitable for long-term tumor immunotherapy. In recent years, hybrid delivery platforms embedding Ad vectors in liposomes or nanoparticles have attracted the interest of researchers, which utilize the advantages of both viral and non-viral vectors to reduce the required dose, improve pharmacokinetics, and enable additional surface modifications as a means of enhancing targeting and immune activation [38,44,45,46,47]. Future research could focus on optimizing the release kinetics of such strategies, improving their intracellular transport, and reducing off-target effects to further enhance the therapeutic capabilities of vaccines.

Ad vectors possess strong immunogenicity, episomal stability, and high transduction efficiency, making them effective platforms for therapeutic cancer vaccines. Capsid engineering and hybrid formulations have expanded their tissue tropism and reduced immunodominance. Ad-based vectors are especially suitable for immunogenic tumors (e.g., melanoma), virus-associated cancers (e.g., HPV-positive cervical cancer), and solid tumors with defined antigens such as prostate cancer [48,49]. Nonetheless, pre-existing neutralizing antibodies, hepatotropism, and systemic toxicity remain barriers. Advanced vector modification and localized delivery approaches are addressing these challenges.

### 2.2. Adeno-Associated Virus (AAV)

AAV is a non-enveloped, single-stranded DNA virus that was first isolated from adenovirus preparations in 1966. It is mainly composed of three viral proteins—VP1, VP2, and VP3 [50,51]. AAV offers multiple advantages as a gene-delivery vector, including high cargo volume, lack of pathogenicity, low toxicity, sustained gene expression, and tissue complement-dependent serotypes [52,53,54]. Since its first use for gene transfer in 1987, AAV has become a central platform for gene therapy research [55,56]. The immunogenicity of AAV is relatively low because the virus is nonpathogenic and latent in the absence of a helper virus such as adenovirus. However, residual epitopes associated with adenovirus may elicit immune recognition that limits repeated dosing [57,58]. Although AAV is currently primarily used for gene therapy, its durable transgene expression ability and inherent safety profile make it useful as a tumor vaccine vector. More importantly, capsid proteins can be rationally designed by genetic engineering or surface modification to improve their targeting to lymphoid tissue or tumor-resident immune cells.

Targeting strategies for AAV vectors can be divided into two main categories: genetic engineering and modification of capsid proteins. Given the limited natural tropism of AAV to tumors, these strategies were designed to enhance delivery specificity and functional expression in the immunosuppressive microenvironment. Among them, the former involves modulation of tissue tropism through amino acid sequence changes or insertion of functional elements, such as TLR9 inhibitory keynotes or PD-1 decoy constructs, to enhance gene expression and immune activation in a specific microenvironment [59,60]. The latter involves the generation of heritable capsid protein variants, the insertion of genes into targeting ligands (e.g., antibodies or peptides), or chemical conjugation to achieve cell-type specificity [61,62,63,64,65].

Although both AAV and Ad show promise for inducing anti-tumor immunity, their efficacy is still constrained by a number of factors, including immunosuppressive elements in the immunosuppressive tumor microenvironment, pre-existing neutralizing antibodies, and differences in the expression of tumor antigens in patients with HLA restriction. To address these limitations, efforts have been made to enhance targeting, stabilize vectors, and stimulate sustained antigen expression in the tumor microenvironment. Through these strategies, the aim is to achieve precision therapy, promote durable T-cell immune responses, and ultimately enhance the therapeutic efficacy of viral vector-based cancer vaccines.

AAV vectors feature low immunogenicity, long-term gene expression, and favorable safety profiles. They are well-suited for prophylactic or low-burden tumors, particularly glioblastoma or early-stage prostate cancer, where durable immune surveillance is required [66,67,68]. While their small size facilitates deep tissue penetration, weak immunogenicity and susceptibility to neutralizing antibodies limit their standalone efficacy. Heterologous prime–boost regimens and immunostimulatory co-delivery can enhance response, and capsid redesign continues to improve targeting. Table 1 summarizes some cases for improving the targeting efficiency of Ad and AAV vectors, including examples of gene reprogramming and combinatorial delivery systems in tumor immunotherapy.

### 2.3. Poxvirus Vectors

Poxviruses are double-stranded DNA viruses that infect vertebrates and insects and comprise at least 46 subfamilies. Of these, varicella virus, the causative agent of smallpox, is best known as the poxvirus [77,78]. The successful development of a poxvirus-based smallpox vaccine played a central role in the global eradication of smallpox [79]. Poxviruses have unique biological advantages for vaccine delivery, including large volumes of foreign genes, natural tissue tropism, and lack of risk of genomic integration [80]. Wild-type poxviruses exhibit intrinsic tumor-selective replication, which can be genetically modified to further improve tumor targeting and immunogenicity [81,82].

Physical and chemical surface modifications are commonly used to enhance targeting. In 2018, Ylosmaki et al. conjugated tumor-specific therapeutic peptides to viral envelopes, which facilitated antigen uptake by APCs and elicited tumor-specific T-cell responses [83]. In 2021, Carlisle’s group at the University of Oxford demonstrated that encapsulation of poxvirus in an amphiphilic polymer shielded it from neutralizing antibodies, followed by targeted delivery of anti-MUC1 antibodies to MUC1-expressing tumor cells by conjugation of anti-MUC1 antibodies to the surface of the ampholymer [84]. Although this change is not inherited, it is technically simple, time-saving, and suitable for time-sensitive personalized clinical applications.

Poxviruses have become an important platform for constructing oncolytic viruses (OV) and mediating oncolytic immunotherapy. Targeted engineering strategies include deletion of toxic genes, such as thymidine kinase (TK), A35, or A49, and insertion of tumor-specific elements to enhance tissue selectivity and antitumor potency [85,86,87,88,89,90,91]. In 2018, Ricordel et al. developed a poxvirus strain with broad tumor infectivity through iterative screening and added a suicide gene (FCU1) along with TK deletion, thereby enhancing tumor targeting [92]. In addition, cell-based delivery systems have been explored to overcome systemic clearance and improve delivery efficiency. A notable example is the “Trojan horse” approach proposed by Draganov and colleagues in 2019, in which oncolytic poxvirus is encapsulated in mesenchymal stem cells (MSCs) and delivered to the tumor site to evade immune surveillance using the tumor-homing ability of the MSCS [93]. A common strategy to enhance the tumor-selective replication of poxviral vectors is to delete the TK gene, the absence of which restricts the ability of poxviruses to replicate in non-dividing normal tissues, while allowing the virus to preferentially replicate in rapidly proliferating tumor cells. The higher transgenic capacity and safety of poxviruses compared to Ad vectors make them ideal platforms for complex immunotherapy. However, the clinical translation of poxviruses faces some limitations, such as host immune memory affecting repeat vaccination and immunosuppressive factors in the immunosuppressive tumor microenvironment leading to reduced vaccine therapeutic efficacy [90]. Current research on poxviruses is focused on enhancing targeting capabilities while combining poxvirus vaccines with checkpoint inhibitors, radiotherapy, or other combination therapies to improve the overall efficacy of vaccines in cancer immunotherapy [94].

Poxviruses provide large genetic capacity and inherent tumor tropism, making them well-suited for encoding multi-antigenic or immunomodulatory payloads. They are particularly promising in tumors with high antigenic heterogeneity, such as renal cell carcinoma and prostate cancer [95,96]. Although effective in heterologous prime–boost or adjuvant settings, challenges including particle size, innate immune activation, and immune memory to the vector limit repeated use. Advances in encapsulation, surface engineering, and delivery integration are helping to improve precision and reduce systemic reactivity.

### 2.4. Oncolytic Virus (OVs)

In addition to the several viruses described above, a variety of other viruses, such as herpes simplex virus (HSV), measles virus (MV), and Newcastle disease virus (NDV), have been engineered as oncolytic vectors that selectively infect tumor cells and initiate antitumor immune responses [97]. Concomitant combination with immune adjuvants or checkpoint inhibitors increases T-cell activation and limits immune escape [98,99,100,101].

Structural modifications of the virus further improved targeting and replication specificity [102,103,104,105]. Tumor-targeting is enhanced by vector surface integration of ligands with affinity for tumor-associated receptors [106,107,108]. Capsid modification improves intratumoral viral delivery by means of tumor-targeting peptides conjugated to the viral surface [83,109,110,111]. Loss of immune-evasion genes has also been shown to enhance T-cell responses by promoting dendritic cell uptake and enhancing antigen presentation [111]. Overall, these approaches improve vaccine selectivity and immunogenicity, supporting the development of oncolytic virus-targeted vaccine platforms.

**Figure 2:**
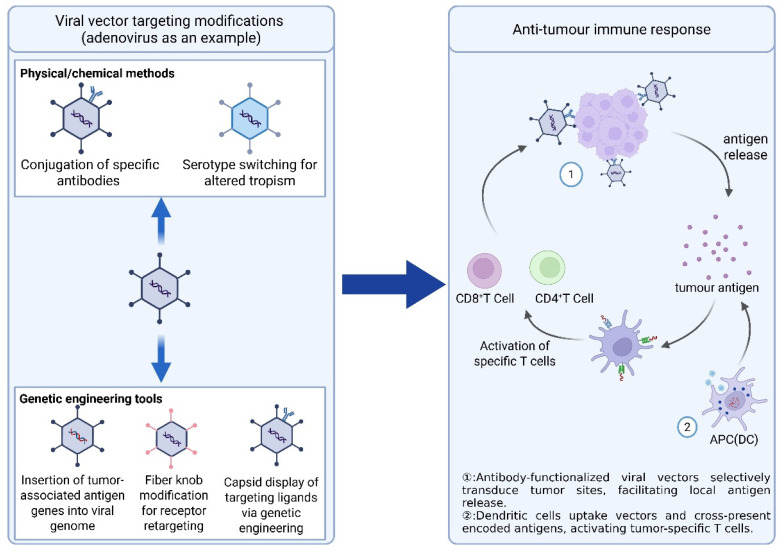
Schematic of vector retargeting strategies and antitumor immune activation mechanisms. Using adenovirus as an example, the figure illustrates engineered tropism and the associated pathways of immune stimulation. Created in https://BioRender.com.

Immune memory against viral vectors, particularly adaptive immunity mediated by neutralizing antibodies, constitutes a major obstacle to repeated dosing, leading to diminished antigen delivery and reduced vaccine efficacy. To reduce the impact of neutralizing antibodies, a heterologous primary immunization-boosting strategy has been developed, whereby different viral vectors are used sequentially to maintain memory and effector T-cell responses. This strategy has been validated in SARS-CoV-2 vaccine studies and is currently being translated into cancer vaccine applications [112,113,114]. In addition, the immune activation strength and specificity of the primary-booster strategy can be further enhanced by improving vector targeting, providing new optimization directions for tumor vaccine design. OVs combine direct tumor lysis with in situ immune activation, functioning as self-amplifying cancer vaccines. They are particularly effective in tumors with impaired antiviral defenses or those amenable to local injection, such as melanoma, glioblastoma, and hepatocellular carcinoma [115,116]. Structural modifications and surface functionalization improve tropism and immune activation. However, their systemic application is hindered by pre-existing immunity, rapid clearance, and limited intratumoral spread. Rational vector engineering, delivery via carrier systems, and combination with immunotherapies may broaden their clinical efficacy. Figure 2 summarizes the engineering strategies and immune mechanisms of viral vectors.

## 3. Non-Viral Vector Vaccines

Non-viral vectors avoid gene integration and treatment risks and provide greater design flexibility and higher biosafety for cancer vaccine development. Non-viral vector platforms can be divided into nanoparticle vectors, cell-based delivery platforms, biofilm-derived vectors, bacterial vectors, and so on [20,117,118,119]. Among them, nanoparticle-based systems have become the core of non-viral delivery of cancer immunotherapy.

### 3.1. Nanoparticle Delivery Systems

#### 3.1.1. Lipid Nanoparticles (LNPs)

Lipid nanoparticles (LNPs) offer enhanced biocompatibility, low toxicity, and improved tissue penetration compared to traditional carriers [120,121]. They have been widely used in RNA-based delivery, such as mRNA COVID-19 vaccines [122,123,124]. Standard formulations consist of phospholipids, cholesterol, polyethylene glycol (PEG)—lipids, and ionizable lipids [125].

The reduced blood flow through the liver and spleen sinusoids leads to the accumulation of LNPs in the liver, thereby limiting extrahepatic delivery [126]. Passive, endogenous, and active targeting strategies have been developed to address this problem [127,128]. Solid lipid nanoparticles (SLNs) loaded with curcumin or carvacrol achieve passive targeting through the enhanced permeability and retention (EPR) effect—a process driven by tumor-induced angiogenesis, in which newly formed vasculature exhibits high permeability and impaired lymphatic drainage, resulting in preferential accumulation of nanocarriers within tumor tissue [129,130,131], but tumor heterogeneity and irregular vasculature compromise precision.

Endogenous targeting is dependent on the pattern of serum protein adsorption, which in turn is regulated by lipid composition [128,132]. A restricted lipid identified by Lokugamage et al. is selectively delivered to splenic T cells [133]. Further adjustments in lipid ratios have improved biodistribution to non-hepatic sites [134,135,136,137,138,139,140,141,142,143,144]. Table 2 lists representative lipid modification methods that can improve tissue selectivity.

Active targeting uses ligand or antibody binding to direct LNPs to specific sites [145,146,147,148,149,150]. Recent studies have developed high-density lipoprotein (HDL)—like LNPs for lymph nodes and antibody-functionalized mRNA-LNPs for HER2-positive (HER^2+^) breast tumors [151,152]. Meanwhile, mRNA optimization techniques such as circular RNA construction can extend translation time, providing new ideas for the development of next-generation LNP tumor vaccines [153,154,155]. However, the LNP platform still faces a number of challenges, including PEG-related pseudoallergy [156], endosomal escape mechanisms that are still not fully understood, and instability during transport [157]. It is worth noting that optimization of vector targeting not only helps to improve the specificity of antigen delivery and immune activation efficiency but also reduces the accumulation in non-specific tissues and thus side effects, which ultimately improves the overall safety and efficacy of the vaccine. Therefore, the combination of active targeting strategies and endogenous targeting strategies (e.g., systemic component modulation) is expected to promote the further development of LNP clinical translation.

Lipid nanoparticles represent a clinically validated, modular platform for RNA-based tumor vaccines. Their favorable biocompatibility, tunable biodistribution, and low immunogenicity have enabled successful mRNA delivery in both infectious and cancer settings. LNPs are particularly effective in melanoma and liver cancers, benefiting from enhanced immunogenicity and hepatic tropism [158,159]. However, challenges such as PEG-induced hypersensitivity, suboptimal endosomal escape, and off-target liver accumulation limit broader application. Combining endogenous lipid tuning with ligand-based active targeting may improve selectivity, reduce systemic toxicity, and expand use across diverse solid tumors.

**Table 2:** Representative LNP targeting strategies.

Targeting Mechanism	Strategy	Specific Approach	Reference
Passive	3-component formulation (3-Comp)	Cholesterol removal enhances pulmonary tropism	[160]
		Cholesterol removal combined with miR-122/142-modified mRNA for dual organ/cell targeting	[161]
	Selective Organ Targeting (SORT)	Addition of charged lipids enables organ-specific tropism	[162]
		Anionic lipids promote splenic accumulation	[163]
	Component replacement	Bile acid substitution for cholesterol enhances splenic targeting	[164]
	Ionizable lipid screening	Lipid library screened for lung-specific delivery	[165]
	Hydrophobic tail optimization	Branched chains increase ovarian tumor selectivity	[166]
	Ionizable lipid + phospholipid tuning	T-cell targeting achieved via phospholipid enrichment and cholesterol reduction	[167]
	pH-responsive lipids	CL4H6 lipid (a synthetic ionizable lipid) enables delivery to tumor-associated macrophages	[168]
Active	Antibody conjugation + chemotactic cue	Surface anti-PECAM-1 and cationic lipid chemotaxis enhance lung targeting	[169]
	Surface peptide conjugation	D-peptide–PEG conjugates direct LNPs to PD-L1^+^ tumor cells	[170]
		Pardaxin-modified LNPs facilitate endoplasmic reticulum (ER)-specific delivery	[171]
	Ganglioside insertion	CD169 targeting enabled via ganglioside incorporation	[172]
	Dendritic cell (DC) membrane coating	DC membrane-coated LNPs target TME	[173]

#### 3.1.2. Polymeric Nanoparticles (PNP)

Polymeric nanoparticles (PNPs) have become ideal platforms for cancer vaccine delivery due to their tunable surface chemistry, biocompatibility, and biodegradability [174,175]. PNPs were first described by Speiser in 1969 and later developed by Robert Langer. Compared with liposomes, the structural and component flexibility of PNPs enables them to better match the physicochemical requirements of the TME, thus enabling passive and active targeting strategies [176,177].

Passive targeting strategies exploit intrinsic properties of the TME, such as acidity and hypoxia, or intrinsic properties of nanoparticles, such as differences in permeability due to particle size and composition [178,179,180]. In 2023, Zhou et al. designed ionizable, pH-sensitive PNPs that can release c-di-GMP (CDG) in acidic endosomes and activate stimulators of interferon gene (STING) signaling to achieve the purpose of tumor treatment [181]. The proportion of polyethylene glycol (PEG) on the surface of polymeric nanoparticles (PNPs) critically modulates their in vivo biodistribution. By tuning PEG density, organ tropism can be selectively altered, thereby enhancing target-site accumulation while reducing off-target toxicities—an essential parameter in optimizing PNP-based vaccine delivery [182,183]. In 2024, Tian et al. used polyvinyl alcohol (PVA)-modified poly (lactic-co-glycolic acid) (PLGA) nanoparticles that were stable in macrophages and responsive to acidic TME to achieve site-specific release and mimic natural delivery, providing a new idea for subsequent research [184].

Active targeted binding of functionalized ligands, such as small molecules or monoclonal antibodies [185,186,187,188,189,190,191,192,193,194,195,196,197,198,199]. In 2024, Wang’s group achieved tumor-targeted delivery in multiple preclinical models using albumin/polyester nanoparticles conjugated to PDL1, 4-1BB, and NKG2A (or TIGIT) antibodies [200]. In addition, genetically engineered modifications of surface ligands or receptors have also been used to enhance binding affinity with receptor-mediated uptake [201].

The development of PNP platforms is currently undergoing a gradual shift from single-targeted strategies to multi-mechanism synergistic delivery. Passive targeting and active targeting are common single-targeting strategies, but passive targeting is limited by tumor heterogeneity and local barriers, compared to active targeting, which is more precise and flexible in design and closer to clinical translational needs. With the advancement of technology, the integration of active and passive targeting strategies is a major trend in optimizing PNP delivery systems. For example, through the use of environmentally responsive polymers to improve spatial and temporal release control, and the superimposition of specific ligands to promote local uptake and immune activation in tumors. Such composite carriers can improve the bioavailability and safety of vaccines in complex tumor environments and are expected to break through existing delivery bottlenecks. Future research should focus on the screening of highly selective targeting proxies and the validation of the composite delivery mechanism to promote the clinical application of PNPs in therapeutic tumor vaccines.

PNP offer versatile physicochemical tunability, surface functionalization, and environmental responsiveness, making them suitable for vaccine delivery in complex TME. They support both passive (e.g., pH/hypoxia-triggered) and active (e.g., ligand-directed) targeting. PNPs are especially effective in tumors with acidic or heterogeneous profiles, such as colorectal and breast cancers [202,203]. Despite these advantages, clinical translation is constrained by tumor heterogeneity, variable release kinetics, and formulation complexity. Future advances should focus on multistage targeting systems integrating environmental triggers and multi-ligand engagement to enhance precision and efficacy. Table 3 below shows the advantages and disadvantages of common polymer particles and their common application ranges.

#### 3.1.3. Inorganic Nanoparticle Carriers

Inorganic nanoparticles, including semiconductors and metal-organic frameworks, are used as cancer vaccine carriers due to their unique physical and chemical properties. These systems typically rely on EPR effects for passive tumor targeting. Some engineered variants show better immunomodulatory capabilities. For example, Cu^2+^-doped titanium dioxide (TiO_2_) nanoparticles have been shown to transiently hyperactivate dendritic cells, thereby enhancing DC uptake and improving antigen presentation [215]. Trail-loaded periodic mesoporous organosilicon (PMO) particles combine passive and active targeting to enhance therapeutic efficacy [216,217,218,219,220,221]. However, high doses of inorganic carriers can easily lead to off-target accumulation and systemic toxicity [222]. The limitations of synthesis uniformity and pore size-dependent degradation further limit the clinical use of inorganic nanoparticles. Compared with organic nanoparticles, inorganic nanomaterials are easier for surface modification and compositional modulation, but non-targeted toxicity at high doses and difficulty in preparation remain barriers to translational applications. Future optimization of inorganic nanoplatforms should take into account immune activation and controlled degradation capabilities, and prioritize targeting and safety dynamics.

**Figure 3:**
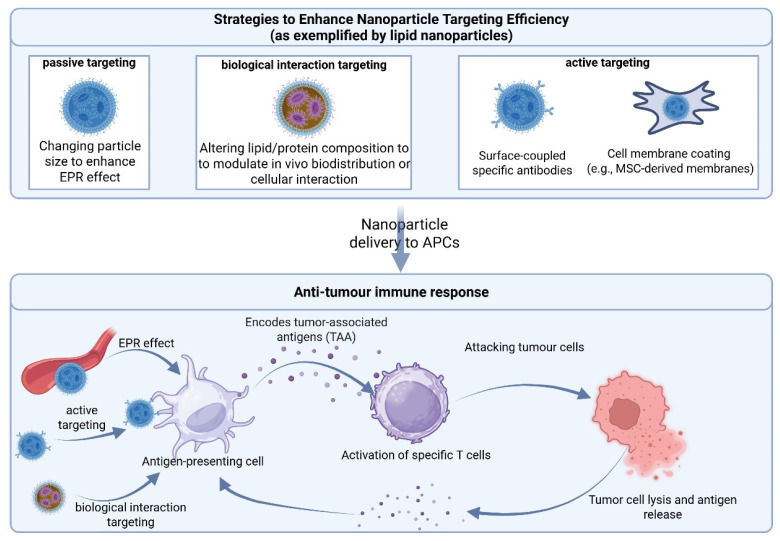
Schematic of nanoparticle carrier retargeting strategies and antitumor immune mechanisms. Using lipid nanoparticles (LNPs) as an example, the figure illustrates design modifications and their functional effects. Created in https://BioRender.com.

Inorganic nanoparticles offer structural rigidity, high surface modifiability, and programmable immune stimulation. Their EPR-based passive targeting and functionalized surface modifications support multi-modal vaccine delivery. These systems are particularly useful in tumors requiring enhanced antigen presentation, such as breast and colorectal cancers [223,224]. However, their clinical potential is limited by synthetic heterogeneity, poor biodegradability, and off-target accumulation at high doses. Prioritizing immune-responsive, degradable nanoscaffolds with controllable release may help address safety and translational challenges. Table 4 summarizes the compositions, sizes, morphologies, key applications, and associated challenges of three representative inorganic nanoparticle carriers. Additionally, Figure 3 summarizes the targeting strategies and mechanisms employed by nanoparticle-based delivery platforms, using lipid nanoparticles as an example.

Nanoparticles are more promising than viral systems, but the complexity of formulation poses challenges for production and purification [232]. While focusing on targeting, clinical feasibility should be considered to avoid off-target effects and inflammation [233].

With the maturity of artificial intelligence-assisted formulation screening and high-throughput functional evaluation systems, nanomaterials are expected to make breakthroughs in precise structural design, tissue selectivity, and immune response modulation and to become important delivery tools for the next generation of therapeutic tumor vaccines.

### 3.2. Cell-Based Delivery Platforms

Cell-based delivery systems leverage vesicle-mediated signaling and surface ligand expression for targeted delivery. In the context of mRNA vaccines, the genetic engineering to express patient-specific surface markers or transport receptors allows precise cancer treatment [234].

#### 3.2.1. Dendritic Cells (DCs)

DCs have been widely used due to their strong antigen presentation ability and high mRNA transfection efficiency [235]. In 2021, Harris et al. enhanced DC activation and improved passive targeting by knocking down AIM2, a type I interferon-induced sensor [236]. However, dendritic cell vaccines are clinically challenged by insufficient T-cell activation, an obstacle that has prompted researchers to shift from passive targeting to strategies that enhance active targeting and co-stimulatory signaling through cell surface functionalization. For example, Yang et al. used copper-free click chemistry and metabolic glycoengineering to functionalize the DC surface, improving DC-T-cell interactions and anti-tumor activity [237]. Targeting strategies for dendritic cell vaccines include both passive and active approaches to optimize T-cell activation and function [238,239]. Clinical translation of DC vaccines is still limited by manufacturing costs, technical complexity, and inter-individual immunophenotypic differences. DC vaccines leverage potent antigen presentation and have demonstrated clinical promise in tumors that require strong T-cell priming, such as melanoma and prostate cancer [9,20]. Their advantages include high mRNA transfection efficiency and ex vivo manipulability, enabling surface engineering to enhance targeting and co-stimulatory signaling. However, challenges such as complex manufacturing, inter-patient immunophenotypic variability, and limited in vivo expansion restrict their scalability. Standardizing off-the-shelf engineered DC platforms with active targeting ligands may help overcome these barriers and broaden their translational potential in solid tumors.

#### 3.2.2. Engineered Immune Cells

Advances in gene editing technology and the success of CAR-T therapies have fueled the progress of engineered immune cells as a delivery platform. Unlike other platforms, immune cells inherently possess some tumor-homing ability. To enhance their specific delivery capacity, researchers have targeted modulation by introducing chimeric antigen receptors or artificial receptors, etc. In 2023, Zhao et al. introduced chimeric antigen receptors into NK cells for targeting B7-H3 in solid tumors [240]. In 2024, Wang et al. added HER2-binding affinities to macrophages to enhance the response to HER2^+^ tumor selective delivery [241]. In addition to exogenously engineered modifications, the tumor-homing ability of immune cells offers additional possibilities for targeted delivery.

Although engineering means can well enhance vector targeting performance, limited autologous cell sources, ex vivo and in vivo expansion efficiency, and individual differences hinder further clinical development. Engineered immune cells, such as CAR-NK cells and macrophages with synthetic receptors, offer both active tumor homing and programmable surface targeting. These vectors are particularly well-suited for solid tumors with defined antigen expression and accessible microenvironments, including HER2^+^ breast cancer and B7-H3^+^ neuroblastoma [242,243]. Despite their advantages, challenges such as limited scalability and patient-to-patient variability restrict widespread use. The development of universal, off-the-shelf immune cell platforms with modular targeting capabilities is a promising direction toward overcoming these barriers and enabling broader clinical translation [244,245,246].

#### 3.2.3. Stem Cells

Stem cells are emerging as a platform for tumor vaccines due to their plasticity, tumor affinity, and wide range of sources [247,248,249,250,251]. For example, Kotlevsky’s group used neural stem cells and their exosomes to deliver vaccines to hypoxic glioma regions [252]. It was shown that their tumor localization ability was further enhanced by surface modification of specific ligands or selection of specific tumor-homing strong cell types [253,254,255,256]. Mesenchymal stem cells (MSCs) and their derived exosomes have shown particular promise in solid tumors with rich stromal components—such as glioblastoma, pancreatic, and breast cancers—where they support efficient cargo delivery and can be engineered to co-deliver immunomodulatory signals or differentiate into dendritic-like cells to enhance immunogenicity [257,258,259]. However, under certain conditions, stem cells may promote tumor development [260]. Moreover, variability in donor sources and the complexity of manufacturing continue to impede standardization. Therefore, cell-based platforms may be most suitable for tumors requiring localized, cell-guided delivery and sustained immune modulation. Future optimization should prioritize the selection of defined subpopulations and the clarification of tumor-tropic mechanisms to improve translational readiness.

### 3.3. Membrane-Derived Vesicular Carriers

Exosome-based delivery systems are derived from biofilms, and their inherent tissue tropism and biocompatibility make them an important delivery platform in tumor immunotherapy [261,262,263,264]. In 2022, Le et al. functionalized erythrocyte-derived exosomes with epidermal growth factor receptor (EGFR)-specific antibodies to target EGFR-positive tumor cells [265]. In 2024, Yu et al. utilized peptide-based surface engineering to direct exosomes to the ER, enhancing the therapeutic response [266]. Exosome-based systems can be further refined by genetic engineering, ligand binding, or encapsulation to improve targeting and payload control. However, large-scale production, structural stability, and translational efficiency are still limitations for large-scale clinical applications [261,264,267,268]. These vectors may be particularly effective for tumors with well-defined surface antigens (e.g., EGFR-overexpressing carcinomas) or elevated ER stress, where targeted delivery improves therapeutic precision [269]. Nonetheless, their heterogeneous content and immunomodulatory potential necessitate rigorous standardization for clinical application.

Membrane-wrapped nanoparticles combine the biological targeting properties of cell membranes with the tunability of synthetic nanocarriers [270]. It shows the characteristics of prolonged systemic circulation, immune evasion, and tissue-specific homing in membrane cells [271,272,273,274,275,276]. For example, tumor-associated macrophage membrane mimetic coatings combined with hyaluronic acid and α4β1 integrin have been used to achieve dual-mode targeting and improve immunogenicity [277]. In addition, the different targeting properties of different biofilms and the potential immunosuppressive or tumor-promoting effects pose great risks. In addition, the complexity and cost of membrane separation and fusion processes limit the wide application of such carriers [278,279].

Membrane-coated vectors are particularly suited for immunosuppressive tumors, such as pancreatic and breast cancers, where extended circulation and immune evasion are essential for effective delivery [280]. Nevertheless, the heterogeneity of membrane sources and the risk of incorporating pro-tumoral signals present significant safety and reproducibility challenges. To advance the application of membrane-derived vectors, future work should prioritize (1) the selection of membrane sources with natural tumor-homing ability for targeting needs, (2) the rigorous assessment of the risk of membrane-mediated immunosuppression, and (3) the development of high-throughput and controllable platforms for the preparation of biofilm-derived vectors.

### 3.4. Plant Virus-Derived Nanoparticles for Cancer Vaccine Delivery

Virus-like particles (VLPs) are multimeric protein assemblies that structurally mimic native viruses or bacteriophages yet lack viral genetic material, rendering them non-infectious [281]. The hollow capsid architecture of VLPs enables encapsulation of diverse cargos, including nucleic acids, peptides, and proteins, making them attractive candidates for precision delivery in cancer vaccine platforms. Their intrinsic immunogenicity further supports their role as self-adjuvanted carriers [282,283,284]. First identified by Baruch S. Blumberg in the 1960s, VLPs have since been exploited in the development of prophylactic vaccines against oncogenic viruses [285].

Among the VLP platforms, plant virus-derived VLPs—commonly referred to as plant virus nanoparticles (PVNPs)—have garnered considerable interest owing to their favorable safety profiles, low production costs, and structural stability [286,287]. Beyond serving as delivery vehicles, PVLPs and PVNPs can act as adjuvants to potentiate immune responses [288,289]. Commonly employed plant viruses include tobacco mosaic virus (TMV), potato virus X (PVX), and cowpea mosaic virus (CPMV), each with distinct structural and immunologic properties [290].

PVNPs exhibit a modular architecture conducive to genetic and surface engineering. For instance, covalent coupling of the immunostimulatory protein S100A9 to CPMV nanoparticles, as reported by Chung et al. in 2021, elicited both prophylactic and therapeutic responses in murine models of pulmonary metastatic melanoma and breast carcinoma [291]. Standard bioconjugation and click chemistry approaches have been employed to install targeting moieties, while surface passivation with inert polymers has been used to minimize nonspecific accumulation [292]. Genetic modifications—such as insertion, deletion, or substitution within the capsid protein—enable fine-tuning of PVNP tropism, although such alterations must preserve capsid self-assembly. In addition, physicochemical parameters, including particle size, surface charge, and hydrophilicity, substantially influence passive targeting behavior. PEGylation has been employed to extend systemic circulation time and enhance in vivo persistence [293,294,295].

While PVNPs offer a unique combination of safety, scalability, and tunable targeting, challenges remain. Capsid self-assembly is sensitive to genetic alterations, limiting the feasibility of aggressive genome engineering strategies. As a result, research has focused on external conjugation of targeting ligands [290]. Furthermore, the presence of plant-derived glycoproteins may pose immunogenicity concerns in human subjects, necessitating thorough clinical evaluation. Despite their relatively low manufacturing costs, progress in clinical translation has been slow, partially due to limited commercial engagement [295]. Given their self-adjuvanted properties and lymphoid trafficking capacity, PVNP-based vaccines promise to enhance immune responses effective in tumors with low baseline immunogenicity, such as pancreatic and ovarian cancers [289,296]. In addition, their favorable safety profile supports repeated administration, making them well-suited for tumor types requiring long-term immune surveillance [297,298]. Moving forward, leveraging plant-based expression platforms for scalable production, optimizing surface engineering for enhanced specificity, and integrating PVNPs into combination immunotherapeutic regimens may accelerate their adoption in the cancer vaccine field.

### 3.5. Bacterial Vectors

The unique immune stimulation, tumor localization ability, and pathogen-associated molecular patterns of bacteria provide promising platforms for cancer vaccine delivery [299,300,301,302,303,304,305]. Its genome is easy to engineer and easy to produce on a large scale—a major advantage for clinical translation. In 2024, Arpaia et al. engineered Escherichia coli Nissle 1917 to enhance its tropism to the TME and jointly transmit neoantigen-specific signals [306]. Nguyen’s group designed Salmonella strains carrying dual payloads that enhanced anti-tumor responses in mouse models [307].

In addition to Escherichia coli [308], various bacterial species have been evaluated for natural or engineered targeting capabilities. Lactobacillus and recombinant Bifidobacteria exploit mucosal adhesion for site-specific delivery [309,310]. Other bacteria, including Listeria monocytogenes [311] and photosynthetic bacteria, are also commonly used bacterial vectors [312,313]. In addition, bacterial extracellular vesicles have also become the target vector of choice because of their unique potential for intercellular signaling and immune activation [314,315,316,317].

Bacterial platforms have advantages over viral vectors in terms of operability, scale-up, and system safety, and engineered bacteria such as Salmonella and Listeria monocytogenes have been shown to have tumor-specific colonization and antigen delivery capabilities. However, some clinical challenges remain. Certain strains, such as Helicobacter pylori, may have an oncogenic risk under certain conditions, and the mechanisms behind this are unclear [318]. In addition, limited understanding of the mechanisms and poor patient compliance have hindered widespread clinical use [319]. Bacterial delivery platforms are particularly advantageous in hypoxic or necrotic tumor regions—such as pancreatic and colorectal cancers—where conventional vectors often fail to penetrate. Moreover, the intrinsic immunostimulatory properties of bacterial components render them well-suited for immunologically “cold” tumors [320,321]. Nevertheless, heterogeneity in colonization efficiency across tumor types and potential safety concerns necessitate rigorous strain selection and platform standardization.

In order to fully exploit the potential of bacterial vectors for therapeutic tumor vaccines, future efforts should focus on (1) eliminating oncogenic factors and improving vector predictability through genetic engineering; (2) developing programmable targeting strategies, such as the addition of specific ligands to the bacterial surface for tumor-specific localization; and (3) generating reliable clinical trial data to support the regulatory process. Among them, rational targeting strategies will help bacterial vector systems overcome current limitations and enhance clinical translation.

## 4. Perspective

The future of therapeutic cancer vaccines lies in multimodal vector delivery platforms that integrate spatial collaborative targeting, immune cell-specific binding, and payload release in response to the TME. For example, Chen et al. applied orthogonal cross-linking chemistry to design vectors that could target multiple immune cell subsets in the TME to improve efficacy [322]. Some of these combination strategies include combining vaccine vectors with immune checkpoint inhibitors and “Trojan horses”, in which specific membrane-coated nanoparticles are used to improve delivery precision while controlling toxicity [323,324].

Increasingly, emerging strategies incorporate multiple design features to integrate active targeting, passive stockpiling, and TME response into a unified system. Precise and long-lasting delivery in response to multiple signals. Triple-combination designs of linker vectors, immunostimulants, and checkpoint blockade are being evaluated for their potential for synergistic delivery and immune enhancement [325,326].

Advances in artificial intelligence and data-driven modeling are accelerating rational vector design. Artificial intelligence-driven modeling supports rapid screening of nanoparticle components, while machine learning facilitates target prediction and optimization of active targeting ligands. The integration of clinical data supports a stratified treatment approach, making the vector targeting strategy more specific and in line with precision treatment [327]. Together, these innovations have facilitated the development of targeting capabilities to advance personalized medicine.

## Figures and Tables

**Table 1:** Targeting strategies for Ad/AAV vectors.

Strategy Type	Modification	Target	Reference
Ad fiber engineering	Ad35 fiber replacement to evade neutralization and improve uptake	Guanylyl cyclase C^+^ gastrointestinal tumors	[69]
Ad5 genetic reprogramming	MelARV expression with ISD domain mutation	Tumors expressing endogenous retroviral antigens	[70]
OAd + HDAd vector combination	CAdVEC platform for complex TME adaptation	Solid tumors	[71]
Heterologous Ad-based prime–boost	ChAd68 priming with VEE-samRNA boost	Tumors bearing personalized neoantigens	[72]
AAV capsid S663 mutation + EV delivery	EV-mediated delivery to bypass immune memory and receptor binding	Melanoma	[73]
Ad–PAMAM + hydrogel formulation	Localized release via hydrogel for enhanced regional accumulation		[74]
Oncolytic Ad + tumor-derived EVs	Trojan horse delivery using tumor membrane camouflage		[75]
Multi-serotype AAV vector	Co-delivery of PD-1 and tumor antigens to dendritic cells	Mesothelioma	[76]

**Table 3:** Comparison of the advantages and disadvantages of common polymers and their common application ranges.

Polymer Type	Advantage	Disadvantage	Common Applications	Reference
PLGA	Biodegradable; good biocompatibility; FDA-approved; controlled release	Acidic degradation products acidify microenvironment; rapid clearance without modification	Laser-activated controlled drug delivery at targeted sites	[204,205,206]
PEG	Reduces protein adsorption/opsonization; verified safety; improves nanoparticle stability and circulation	Non-biodegradable, accumulates; antibody induction upon repeated use; cannot form nanoparticles alone	Copolymer to enhance carrier circulation and stability	[206,207]
Chitosan (natural polycation)	Biodegradable; mucosal absorption; strongly binds negatively charged drugs	Batch-to-batch variability; high solubility only under acidic conditions, limiting systemic administration	Mucosal vaccine delivery; systemic vaccine adjuvant	[204,208,209]
Polycaprolactone (PCL)	Biodegradable; good biocompatibility; FDA-approved; efficiently encapsulates hydrophobic drugs	Slow degradation; poor hydrophilic drug encapsulation; forms semi-crystalline matrices, delaying release	Targeted drug delivery (active/passive)	[204,210]
Albumin (protein polymer nanoparticles)	Biocompatible; biodegradable; clinically established; low immunogenicity; tumor-targeting capability	Instability (easy dissociation); biological sourcing complicates purification; limited control over size/drug loading	Delivery of chemotherapeutics (e.g., paclitaxel)	[211,212,213,214]

**Table 4:** Comparison of composition, size, morphology, key applications, and challenges of common inorganic nanoparticles.

Nanoparticle Type	Composition, Size, Morphology	Key Applications	Challenges	Reference
Gold Nanoparticles (AuNPs)	Gold core stabilized by surface modifications; spherical (5–100 nm) or tunable morphologies (rods, shells) with adjustable optical properties	Photothermal therapy, drug/gene delivery, imaging	Non-degradable; immune activation; high production cost	[225,226]
Mesoporous Silica Nanoparticles (MSNs)	Amorphous silica with ordered nanopores; spherical (~50–200 nm), pore diameter 2–6 nm; usually surface-modified	Sustained-release drug delivery, combination therapy	Residual additives; liver accumulation; poor biodegradability; mechanical brittleness	[227,228,229]
Iron Oxide Nanoparticles (Magnetic NPs)	Crystalline iron oxide stabilized by coatings; spherical (5–50 nm); ≤20 nm particles show superparamagnetism (no residual magnetization)	Magnetic targeting, hyperthermia, MRI imaging contrast agents	Coating-dependent stability; limited targeting depth; dose-related toxicity	[230,231]

## Data Availability

Not applicable.
